# P-499. Evaluation for congenital syphilis at a single center in Utah from 2014-2023

**DOI:** 10.1093/ofid/ofaf695.714

**Published:** 2026-01-11

**Authors:** Brent D Nelson, Sonia Mehra, Shelley M Lawerence

**Affiliations:** University of Utah, Salt Lake City, UT; University of Utah, Salt Lake City, UT; University of Utah School of Medicine, Salt Lake City, Utah

## Abstract

**Background:**

Historically, Utah has had a very low incidence of congenital syphilis (CS). Infants at risk for CS but not meeting the Centers for Disease Control and Prevention case definition, are not tracked by the Utah Health Department. Identifying all at-risk infants more fully describes the impact of CS in pediatrics and may be an early indicator of epidemiological trends. Understanding the management of infants at risk for CS clarifies opportunities to improve care.

**Figure:**
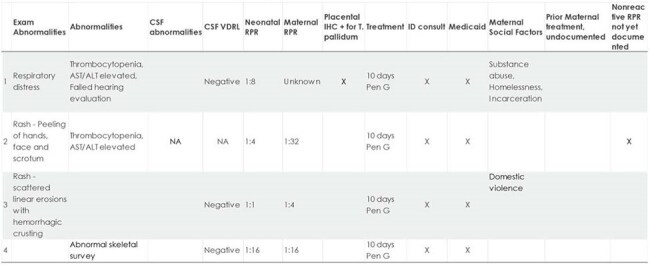
4 Infants with Proven/Highly Probable CS

**Figure:**
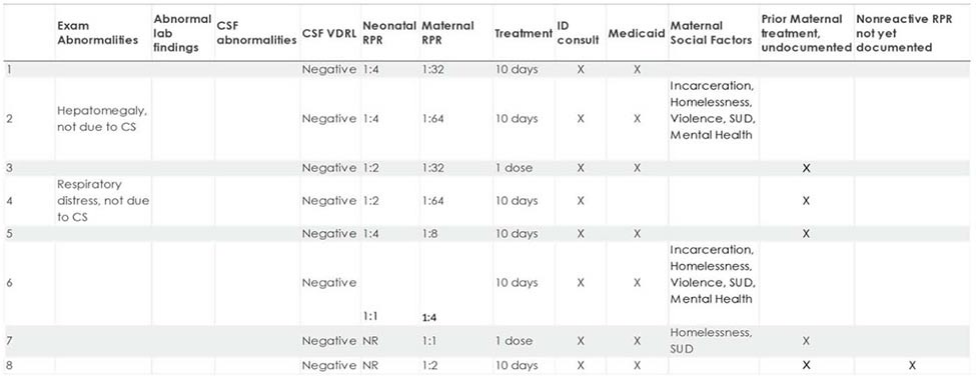
8 Infants with Possible CS

**Methods:**

This is a retrospective review of infants at risk for CS from 2014 to 2023 at the University of Utah. Infants included had a rapid plasma reagin (RPR) test drawn within the first year of life and corresponding diagnosis of syphilis in the mother. Forty-two of 85 infants were excluded with no maternal diagnosis of syphilis or non-Utah resident status. We collected exam, lab, treatment, and socioeconomic factors of infants and mothers. Infants were divided into the American Academy of Pediatrics Red Book categories of “proven/highly probable”, “possible”, “less likely” or “unlikely”.

**Figure:**
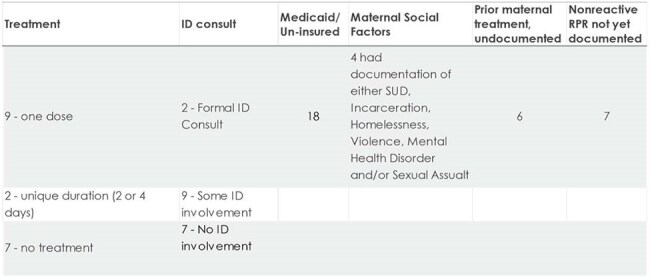
Summary of 19 Infants with Less Likely CS

**Figure:**
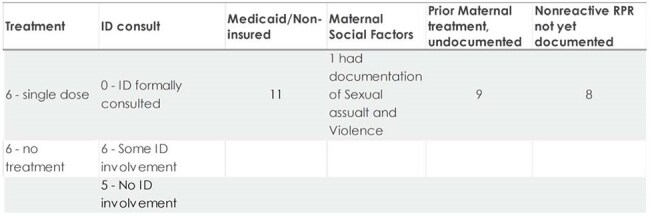
Summary of 12 Infants with Unlikely CS

**Results:**

Of 43 infants identified, 41 were born to mothers with at-risk socioeconomic factors. Infants evaluated for CS increased exponentially from 2014 to 2023. There were 4 "proven/highly probable", 8 "possible", 19 "less likely" and 12 "unlikely" CS cases. Of those in the "possible" category, five mothers had no documentation of prior treatment. Despite being considered "less likely" and "unlikely" to have CS, 15 mothers in these categories lacked clear documentation of treatment. Twenty-one infants were lost to follow up without a nonreactive RPR test.  One infant had laboratory confirmation of CS and bilateral hearing loss. A reactive infant RPR test result was not predictive of disease severity.

**Conclusion:**

Congenital syphilis preferentially impacts those with socioeconomic risk factors. Enumerating infants at risk for CS may predict future trends. Laboratory confirmed CS is rare. Many infants at risk for CS have no laboratory findings or symptoms of infection. Many infants treated as “less likely” and “unlikely” had inadequate documentation of maternal treatment. Although overestimated, the number of infants without adequate follow-up and repeat RPR testing is unacceptably high representing an important opportunity for improvement.

**Disclosures:**

All Authors: No reported disclosures

